# Do Patterns of Adolescent Participation in Arts, Culture and Entertainment Activities Predict Later Wellbeing? A Latent Class Analysis

**DOI:** 10.1007/s10964-024-01950-7

**Published:** 2024-03-11

**Authors:** Emma Thornton, Kimberly Petersen, Jose Marquez, Neil Humphrey

**Affiliations:** 1 Manchester Institute of Education, University of Manchester, Manchester, UK; 2 School of Education, University of Leeds, Leeds, UK

**Keywords:** Arts, Culture, Entertainment, Latent class analysis, Wellbeing, Adolescence

## Abstract

**Abstract:**

Participation in arts, culture, and entertainment (PACE) activities may promote adolescent wellbeing. However, little is known about how such activities cluster together, and previous research has used small samples, cross-sectional designs, focused on single activities, and/or has not considered the influence of socio-demographic factors on participation. Using latent class analysis, the aims of this study were to establish: (i) classes of adolescent PACE activities; (ii) associations between socio-demographic characteristics and PACE classification; and, (iii) whether PACE classification predicts later wellbeing. Longitudinal data from the #BeeWell study (*N* = 18,224 adolescents; mean age at T1 = 12 years 7 months (±3.56 months); 50.54% female) were analyzed. Four latent classes were established: the ‘Dynamic Doers’ (high, wide-ranging participation; 11.87%); the ’Mind and Body Crew’ (reading, arts, videogames, sports/exercise; 39.81%); the ‘Game and Gain Squad’ (videogames and sports/exercise; 29.05%); and the ‘Activity Free Adolescents’ (uniformly low participation; 19.27%). Associations between socio-demographic characteristics and PACE classification were observed (e.g., socio-economic disadvantage increased the likelihood of Activity Free Adolescents classification, compared to Game and Gain Squad classification). Finally, PACE classification predicted later wellbeing (e.g., Dynamic Doers reported significantly higher wellbeing than Activity Free Adolescents). These findings are discussed in relation to the need to improve accessibility and appeal of arts, culture, and entertainment provision for adolescents as a means to optimize their wellbeing.

**Pre-registration:**

The analysis plan for this study was pre-registered on the Open Science Framework and can be found here: https://osf.io/2jtpd

## Introduction

Mental wellbeing, from the perspective of adolescents themselves, is about feeling good (hedonia) and functioning well (eudaimonia) (Deighton, 2016). It is an essential indicator in population health research (Thapar et al., 2021), given the fact that most adolescents do not meet the diagnostic criteria for mental disorder (NHS Digital, 2022), and the enduring recognition that health is about more than the absence of illness (Schramme, 2023). Furthermore, adolescent mental wellbeing is a key marker for school attainment, adult wellbeing, mental and physical health, labor market/socioeconomic, and relational outcomes (Goodman et al., 2015). Recent research indicates that it declined globally between 2015 and 2018 (Marquez & Long, 2021), and has been declining in the United Kingdom for over a decade (The Children’s Society, 2020). Given this, improving understanding of the determinants of adolescent mental wellbeing is a research and policy priority (Mei et al., 2020). The current study focuses on the influence of participation in arts, culture and entertainment activities, directly addressing priorities for future research identified in a recent review (Bone & Fancourt, 2022), and responding to an earlier call for more robust approaches in an area where the available evidence is “generally weak” (Bungay & Vella-Burrows, 2013, p. 44). Specifically, the current study applies person-focused statistical techniques to determine patterns of participation in arts culture and entertainment, determinants of these patterns of participation, and associations between patterns of participation and later mental wellbeing.

The aims of the study are thus to (a) enhance understanding of patterns of adolescent participation in arts, culture, and entertainment; (b) determine the extent to which patterns of participation in arts, culture, and entertainment vary by socio-demographic and other characteristics (e.g., socio-economic deprivation, social media use); and, (c) examine whether patterns of participation in arts, culture, and entertainment predict later wellbeing. The setting of the study is Greater Manchester, UK. This city-region is home to a very diverse population of 2.8 million people, of whom a larger than (national) average proportion are under 16 (Greater Manchester Combined Authority Research, 2023). It has a rich, varied and distinctive arts, culture, and entertainment infrastructure, with an international reputation for ‘doing things differently’ (Greater Manchester Combined Authority, 2019), making it an ideal setting to address the aims outlined above.

Data pertaining to participation in arts, culture, and entertainment activities were collected in the aftermath of the Covid-19 pandemic, between September and December 2021. Although most restrictions had been lifted by this time (Institute for Government Analysis, 2021), there was a slow return to normality in England (ONS, 2022), meaning these data reflect a period of somewhat more limited levels of arts and cultural activity than is typical. Covid-19 restrictions, such as the closure of venues, nationwide lockdowns with stay-at-home orders, and restrictions on large gatherings (Brown et al., 2021), may also have resulted in increases in home-based arts, culture and entertainment activities, such as playing videogames, reading, and/or arts and crafts. It is important to consider engagement in arts, culture, and entertainment activities reported in the current study in this context.

### Adolescent Participation in Arts, Culture and Entertainment (PACE) Activities

Arts, culture and entertainment activities represent key constituent components of leisure. Leisure refers to how free time is spent, and encompasses a wide range of activities undertaken for enjoyment that include (but are not limited to) taking part in hobbies; socializing; volunteering; exercising; and, shopping (Fancourt et al., 2021). The Multi-level Leisure Mechanisms Framework proposes a range of psychological, biological, social and behavioral processes spanning individual, group, and societal levels that interact to produce beneficial effects on physical and mental health (Fancourt et al., 2021). For example, engagement in leisure activities is theorized to promote wellbeing because it fulfils both basic (e.g., sensation, stimulation) and growth (e.g., mastery, relatedness) needs (Sirgy et al., 2017); supports effective emotional regulation and coping; enhances vitality; and, contributes to the development of self-identity, self-acceptance, and social connectedness (Bone & Fancourt, 2022). With specific reference to adolescence, the focus of the current study, these are of course fundamental developmental processes (Christie & Viner, 2005), suggesting that leisure may have particular utility for wellbeing during this period.

Reflecting the renewed interest in the utility of arts and cultural participation (Bone & Fancourt, 2022), the current study focuses on participation in arts, culture, and entertainment in particular, because despite such activities being enshrined in international legislation on children’s rights (e.g., United Nations Convention on the Rights of the Child, Article 31; 1989), funding for arts and cultural engagement has declined in recent years (Easton & Di Novo, 2023). Provision in the education system has waned in parallel, with reductions in the number of arts-specialist teachers, in the time spent teaching arts-based subjects (Ofsted, 2023), and in the proportion of adolescents electing to study them in secondary and further education (Evennett, 2021). The recent Covid-19 pandemic exacerbated this situation, partly because of its impact on access to certain forms of arts, culture, and entertainment during the periods of lockdown (and latterly, social distancing; see above), but also because governmental focus in the ensuing recovery period has prioritized traditional academic learning, with considerably less emphasis on making up for lost opportunities for arts and cultural engagement (Bradbury et al., 2021).

Sports/exercise and entertainment activities are also central to adolescent leisure. Playing games on a computer or console is an increasingly popular form of entertainment, with 91% of young adults (aged 16–24) reporting engagement with some form of gaming (GWI, 2020), and 31.2% of 12–18 year-olds reportedly spending ≥3.5 h a day playing videogames (Skripkauskaite et al., 2022). The prevalence of (online) videogaming may have increased during the aforementioned period of COVID-19-related restrictions, providing an important source of social connection when schools were closed and young people were largely confined to their homes (Pallavicini et al., 2022). With regard to sports/exercise, it is recommended that young people engage in at least 60 minutes of moderate-to-vigorous intensity physical activity on a daily basis (UK Chief Medical Officers, 2019). Importantly, such activity can promote mental health in addition to the more obvious physical health benefits (Panza et al., 2020). The Covid-19 pandemic had a clear impact on this aspect of adolescent leisure, with an estimated reduction of c.20% in total daily physical activity (Neville et al., 2022). As noted above, it is important to consider the data reported in this study in this context.

### Participation in Arts, Culture, and Entertainment and Adolescent Wellbeing

There is a substantial body of epidemiological evidence on the association between participation in arts, culture, and entertainment activities and wellbeing (Adachi & Willoughby, 2017; Bone & Fancourt, 2022; Fancourt et al., 2023; Mahindru et al., 2023). However, a common range of limitations currently restrict the inferences that can be drawn from such research. These include the use of small samples that are not representative of the general population; cross-sectional (as opposed to longitudinal) designs that do not allow temporal precedence to be established; very short follow-up periods when longitudinal designs are implemented; and, failure to account for the influence of socio-demographic differences on participation in arts, culture, and entertainment activities, leading to bias (Bone & Fancourt, 2022). Further, much of the research in this area focuses on adults, with much less evidence pertaining to children and adolescents (Fancourt et al., 2023). The limited number of adolescent studies provide useful insights, but are also illustrative of the above concerns. For example, small, non-representative samples abound (e.g., Trainor et al., 2010), and even though more recent studies using much larger samples have consistently established significant associations between particular arts, culture, and entertainment activities and indicators of wellbeing, these have primarily used cross-sectional designs (e.g., Cosma et al., 2021). When longitudinal designs *have* been implemented, the relationship between participation in arts, culture, and entertainment activities and indicators of adolescent wellbeing appear less clear. For instance, analysis of data from the National Longitudinal Study of Adolescent to Adult Health found that arts engagement was not associated with concurrent or subsequent loneliness; by contrast, undertaking arts activities *did* significantly predict perceived social support one year later, even after adjusting for covariates and previous social support (Bone et al., 2023).

### Person-Focused Approaches to Understanding Adolescent Leisure Time Use

This study adopts a person-focused approach called latent class analysis (LCA), which is used to identify qualitatively different subgroups (classes) within a sample based on responses to a set of observed indicators (Nylund-Gibson & Choi, 2018). LCA is particularly suited to the study of participation in arts, culture, and entertainment as there is currently, “no real consensus on how activities cluster together” (Fancourt et al., 2021, p. 330), meaning that little is known about the heterogeneity of engagement among young people, and consequently the extent to which this is associated with later differences in wellbeing (Sauerwein & Rees, 2020). This is an important evidence gap given the underpinning assumption that engagement in a variety of arts, culture, and entertainment activities (Bone & Fancourt, 2022), or in those which are more meaninguful or fulfilling (Cosma et al., 2021), may be more important for wellbeing than the total time spent on specific activities. LCA can provide a window into both of these aspects. In the current study, the probability of endorsing high frequency of engagement in a given activity is available for each class (time spent). Simultaneously, the range of activities for a given class where there are high probabilities of endorsement can be reviewed (variety). This contrasts with the predominant variable-centered approaches (e.g., multiple regression), which offer benefits in terms of parsimony, but provide minimal specificity as the entire sample is described collectively in terms of the relationship between one variable and another (Howard & Hoffman, 2017).

The small number of existing person-focused studies of leisure time illustrate the utility of this approach. In the only published example involving young people, latent profile analysis of the Children’s World’s dataset was used to identify common patterns of out of school activities. Seven groups were identified (denoted as *very infrequent homework*; *infrequent homework and reading*; *low users of TV, music and computers*; *low media use but high organized leisure*; *highly organized time*; *less organized time*; and, *average*), before establishing associations with socio-demographic characteristics and wellbeing outcomes. For example, the *very infrequent homework* and *infrequent homework and reading* groups were more likely to be male and socio-economically disadvantaged than others, and also reported lower than average life satisfaction. By contrast, the *highly organized leisure time* group were more likely to come from less deprived backgrounds, and reported higher than average life satisfaction (Sauerwein & Rees, 2020). Relatedly, a LCA of the Understanding Society dataset items pertaining to sports, arts, and heritage activities identified six classes of adult cultural engagement (*disengaged*; *low-engagement*; *recreational*; *institutional and historic*; *sports*; and, *omnivore*). Associations between socio-economic position and levels of cultural engagement were observed, with the *disengaged* and *low-engagement* classes more likely to be disadvantaged, and the classes representing more diverse engagement (e.g., *omnivore*) more likely to be advantaged, across a range of indicators (e.g., income, savings, house value) (Walker et al., 2022). Collectively, these studies illustrate the value of person-focused approaches to capture heterogeneity in how leisure time is used, and show how patterns of activity are influenced by sociodemographic characteristics and predict salient wellbeing outcomes such as life satisfaction.

## The Current Study

Participation in arts, culture, and entertainment activities may promote adolescent wellbeing. However, little is known about how such activities cluster together, and previous research has predominantly used variable-focused approaches, small samples, and cross-sectional designs, has focused on single activities, and/or has not considered the influence of socio-demographic factors on participation. The current study addresses these limitations by applying person-focused statistical techniques as part of a longitudinal analysis of a very large dataset using two annual data points (T1, T2) to address the following research questions: What patterns (latent classes) of participation in arts, culture and entertainment activities are identified among adolescents at T1? (Research Question 1); What are the associations between these patterns and social media use, sexuality and gender identity, ethnicity, socioeconomic deprivation, and concurrent mental wellbeing at T1? (Research Question 2); and, Are patterns of PACE at T1 associated with mental wellbeing at T2? (Research Question 3).

## Methods

### Design and Sample

This secondary analysis draws on the first (T1) and second (T2) annual data points of the longitudinal cohort of the #BeeWell study sample (overall *N* = 20,363 from 157 secondary schools across Greater Manchester, England), who were in Year 8 (i.e., aged 12–13) at the first data point. All participants in the #BeeWell longitudinal cohort who completed the full version of the survey (as opposed to the alternative short or symbol versions of the survey^1^; *N* = 20,154 pupils from 149 schools; 99% of overall sample), and responded to at least one of the 11 PACE items noted below (*N* = 18,252; 90% of overall sample) at T1 were considered. As data were clustered by school, schools with 5 or fewer respondents were excised, based on guidance on working with multi-level data (Newsom, 2019)^2^. This led to a total of 18,224 adolescents (89% of overall sample; mean age at T1 = 12 years 7 months (±3.56 months); 50.54% female) from 138 schools being included in the analyses. Analysis of the composition of this analytical sample indicated that it closely mirrored the 11–16 population of adolescents in Greater Manchester and nationally, though with some differences in ethnic composition for the latter (somewhat higher proportion of Asian and lower proportion of White adolescents than across England) (see Table S1, Supplementary Materials).

### Measures

PACE activities and covariates were drawn from T1 survey data and linked administrative data pertaining to ethnicity and socio-economic deprivation. The key response variable, mental wellbeing, was drawn from T2 survey data.

### Participation in arts, culture, and entertainment activities (latent class indicators)

Participants reported their frequency of participation in 11 arts, culture, and entertainment activities when not in school, which were adapted from the Millennium Cohort Study (Center for Longitudinal Studies, 2020). These items were used as indicators for the latent classes (Research Question 1). Possible responses were 1) Never or almost never; 2) Once a year or less; 3) Several times a year; 4) At least once a month; 5) At least once a week; and, 6) Most days. These items were dichotomized into high (most days, at least once a week, at least once a month) and low (several times a year, once a year or less, never/almost never) frequency for use as latent class indicators

The 11 PACE items were:

1. Go to the cinema or theater (24% high frequency; *N* = 4331).
2. Go to watch live sport (22% high frequency; *N* = 4068).
3. Sing in a choir or play in a band or orchestra (9% high frequency; *N* = 1646).
4. Read for enjoyment (not for school) (48% high frequency; *N* = 8672).
5. Go to youth clubs, scouts, girl guides or other organized activities (30% high frequency; *N* = 5523).
6. Go to museums or galleries, visit a historic place or stately home (12% high frequency; *N* = 2123).
7. Attend a religious service (25% high frequency; *N* = 4557).
8. Draw, paint or make things, not at school (50% high frequency; *N* = 9091).
9. Play games on a computer or games console, such as Wii, Xbox, or PlayStation (81% high frequency; *N* = 14,683).
10. Play sports, do exercise, or other physical activities, not in school (80% high frequency; *N* = 14,645).
11. Spend time on creative hobbies, not mentioned above (67% high frequency; *N* = 12,154).

#### Gender identity/sexual orientation (covariate)

Adolescents who identify as belonging to gender and/or sexual minority groups (i.e., lesbian, gay, bi/pansexual, transgender, or questioning; LGBTQ+) experience increased exposure to a range of stressors (e.g., victimization, discrimination, feeling unsafe in the local area; Black et al., 2023) in the context of a cisheteronormative culture (i.e., that which privileges being cisgender and heterosexual; Marquez et al., 2023), which may have the consequence of making certain forms of PACE unappealing and inaccessible. Three categories were derived for this covariate: Cisgender heterosexual boys (33.62%, *N* = 6126); Cisgender heterosexual girls (29.28%, *N* = 5336); and, LGBTQ+ (29.99%, *N* = 5466). The LGBTQ+ group included those who identified as sexual minorities (e.g., gay, lesbian, bi/pansexual), gender diverse (e.g., non-binary, and/or self-reported gender is incongruent with sex assigned at birth, such as those born a boy who identify as a girl), or who indicated that they *describe themselves in another way* or *prefer not to say* on either the sexual orientation or gender identity survey items. Cisgender heterosexual boys and girls were those whose sex assigned at birth and gender were congruent with one another (e.g., born a boy and identify as a boy) and sexuality was reported as heterosexual. Considering the data in this way minimized attrition due to item level missingness in the gender identity and sexual orientation items (i.e., if a respondent is classified as LGBTQ+ based on their sexual orientation response, missing gender identity information is inconsequential, and vice versa).

#### Ethnicity (covariate)

Ethnicity was included as a covariate in light of evidence of disparities in levels of engagement in PACE activities. For example, the aforementioned analysis of adult sports, arts and heritage activities indicated that adults from ethnic minority groups were significantly more likely to belong to the *disengaged* class than their White counterparts (Walker et al., 2022). Ethnicity was derived from linked administrative data provided by Local Authorities, as follows:

- White (65.1%, *N* = 11,864)
- Asian (17.15%, *N* = 3126)
- Black (4.87%, *N* = 887)
- Mixed (5.75%, *N* = 1047)
- Any Other Ethnic Group (including Chinese^3^) (3.18%, *N* = 579)

‘Unclassified’ ethnicity was treated as missing data in our analyses.

#### Socio-economic disadvantage (covariate)

Socio-economic disadvantage was included as a covariate given evidence that engagement in PACE activities is higher among those from more affluent areas among adults and young people outside of school (Mak et al., 2020; Mak & Fancourt, 2021). The indicator used in the analyses was derived from linked administrative data on eligibility for free school meals (FSM) provided by Local Authorities, and linked publicly available data on Index of Multiple Deprivation (IMD) scores.

IMD is a measure of relative neighborhood deprivation formed from data on seven domains (income, employment, health deprivation and disability, education, skills and training, crime, barriers to housing and services, and living environment). Based on an individual’s postcode, these are used to rank small areas (Lower Layer Super Output Areas, which contain ~1500 residents) from the least deprived to the most deprived (Mclennan et al., 2019). FSM eligibility (yes/no) is based on a variety of income-related indicators (e.g., annual gross income of less than £16,190, receipt of Income Support or income-based Job Seekers Allowance) and is thus considered a proxy indicator of low family income.

The current study adopted the approach taken in a recent study utilizing #BeeWell data (Black et al., 2023) to derive a binary measure of socio-economic disadvantage which combined IMD and FSM data, so that a measure of the most disadvantaged young people was obtained (especially given that not every individual living in a deprived IMD area necessarily experiences high levels of deprivation). Specifically, those in the lowest IMD quintile *and* also eligible for free school meals were considered to be disadvantaged (*N* = 2021; 11.09%).

#### Social media use (covariate)

Social media use was included as a covariate in light of research which indicates that increased screen time (including social media use) displaces other activities (Auhuber et al., 2019). The question used in the current study, adapted from the Millennium Cohort Study (Center for Longitudinal Studies, 2020), was: “On a normal weekday during term time, how much time do you spend on social media? For example, sites or apps like TikTok, Instagram, and Snapchat?”. Response options were hourly increments spanning: (1) none; (2) less than 1 h; (3) 1 h to less than 2 h; (4) 2 h to less than 3 h; (5) 3 h to less than 4 h; (6) 4 h to less than 5 h; (7) 5 h to less than 6 h; (8) 6 h to less than 7 h; and (9) 7 h or more. Responses were recoded into a quasi-continuous variable whereby a higher value denoted more daily time spent on social media.

#### Mental wellbeing (covariate at T1; outcome variable at T2)

Mental wellbeing was measured using the Short Warwick-Edinburgh Mental Wellbeing Scale (SWEMWBS; Clarke et al., 2011), which is a 7-item scale (sample item: “I’ve been feeling useful”) with responses as follows: (1) None of the time; (2) Rarely; (3) Some of the time; (4) Often; and (5) All of the time. Consistent with guidance from the measure developer, transformed SWEMWBS scores were used (Stewart-Brown et al., 2009). SWEMWBS has previously been found to exhibit favorable psychometric properties (e.g., good internal consistency, convergent validity, construct validity, and discriminant validity; Ringdal et al., 2018) and was recently identified as the optimal wellbeing measure in the #BeeWell survey in a comprehensive analysis spanning assessment of dimensionality, measurement invariance, and convergent validity (Black et al., 2023). The average T1 wellbeing score was 21.76 (±4.93) and at T2 was 21.78 (±5.04). In both cases, possible scores ranged from 7 to 35, with higher scores indicating better wellbeing.

### Analytic Strategy

All analyses were pre-registered on the Open Science Framework (OSF project osf.io/weshu; Registration DOI 10.17605/OSF.IO/2JTPD) and were conducted using RStudio (Posit Team, 2023) and MPlus version 8.8 (Muthén & Muthén, 1998–2017). Analysis syntax is available at the OSF link above.

Exploratory LCA was used to identify distinct patterns of participation in PACE activities, using the 11 dichotomized T1 PACE items as the latent class indicators (Research Question 1). ‘TYPE = COMPLEX’ was used to account for any effects of clustering by school. A 1-class model was estimated first, followed by models with an increasing number of classes until convergence problems were encountered. The optimal model was selected based on a range of fit statistics and substantive criteria. Information criteria (Akaike Information Criterion [AIC], Bayesian Information Criterion [BIC] and sample-size adjusted Bayesian Information Criterion [ssaBIC]) were compared, with smaller values indicating better model fit. Further, the Lo-Mendell-Rubin adjusted likelihood ratio tests (LMR-LRT) was used to assess whether any given model was a significantly better fit (*p* < 0.05) compared with a model with one class fewer. As fit statistics often do not converge on a single model (Nylund-Gibson & Choi, 2018), the following substantive criteria were also considered: parsimony (i.e., capturing heterogeneity in as few classes as possible); distinctiveness (i.e., the selection of models where classes were distinct from each other); and, class size (i.e., any models with classes that contain a very small proportion of the sample were excluded, as they may indicate overextraction or unstable classes; Masyn, 2013). A split-halves analysis was also conducted, in which the analytical sample was randomly split in half, and the latent classes were re-estimated in each. PACE item endorsement probabilities in both halves of the sample were compared to determine whether endorsement was similar (see Tables S2, S3, and S4, Supplementary Materials). This step of the analysis was not included in the pre-registered analysis plan, but was added to increase the rigor of the class enumeration procedure.

**Fig. 1:**
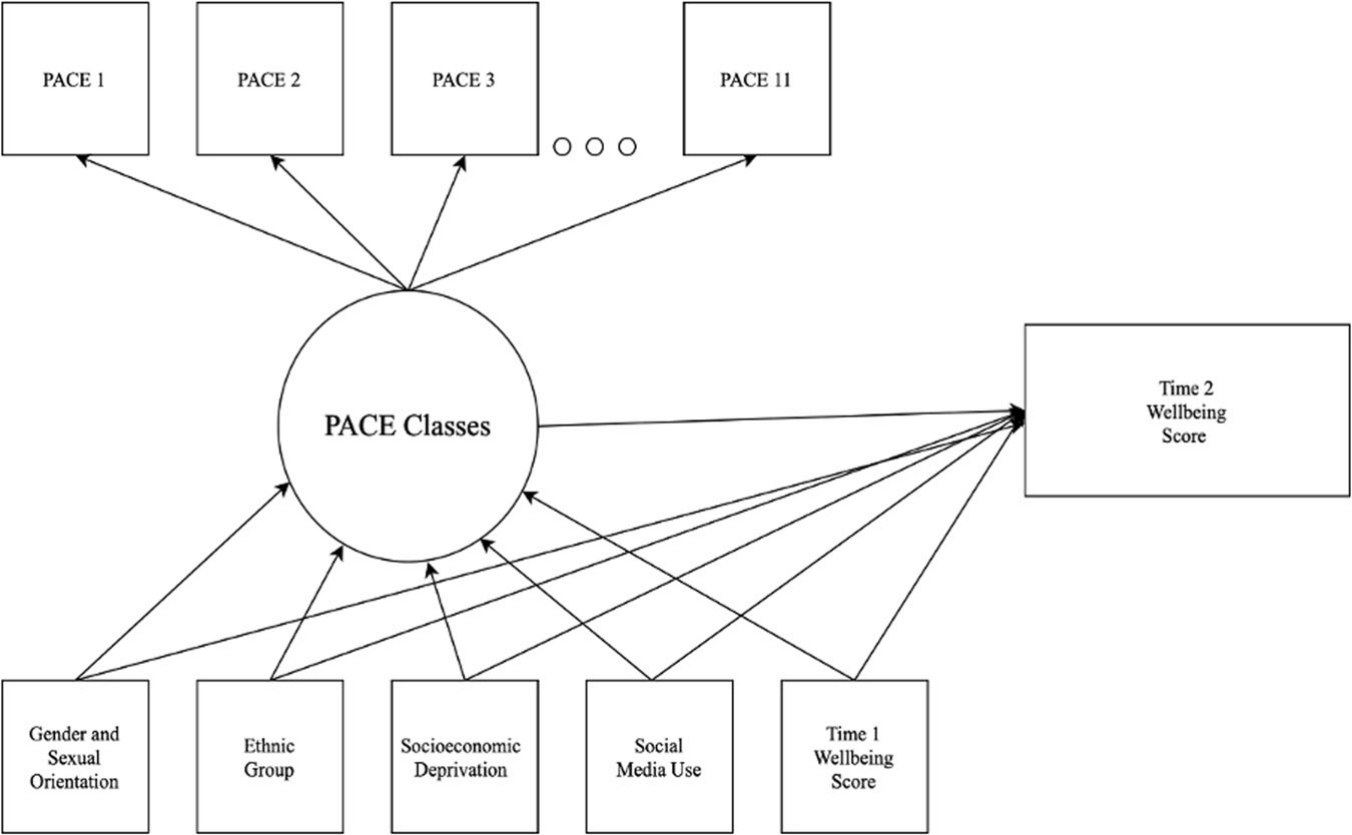
Conceptual diagram showing the latent class model with covariates and distal outcome

Once the best fitting model was selected, a latent class regression analysis was conducted to investigate associations between PACE classes and covariates, i.e., T1 social media use, sexuality and gender identity, ethnicity, socioeconomic deprivation, and concurrent mental wellbeing (Research Question 2), and mental wellbeing at T2 (Research Question 3). Incorporating covariates and/or distal variables in the latent class model in a single step is problematic because the covariates can influence class formation, changing their original meaning (Asparouhov & Muthén, 2014). To overcome this issue, the manual Maximum Likelihood (ML) three-step method (Nylund-Gibson & Choi, 2018) was used. This involves: (1) identifying the optimal number of latent classes; (2) saving the most likely class membership along with classification error; and, (3) carrying out regression analyses with the saved classes whilst taking misclassification into consideration. A latent class regression model which regressed the covariates on PACE classes and the outcome variable (T2 mental wellbeing scores; see Fig. 1 for conceptual diagram) was fitted. This analysis, which adjusted for Time 1 covariates (gender and sexual orientation; ethnicity; socioeconomic deprivation; social media use; and, T1 wellbeing scores) enabled estimation of the extent to which patterns of PACE predicted wellbeing at T2 by contrasting these outcomes across the latent classes (Research Question 3). In addition, an unadjusted sensitivity analysis where PACE classes were regressed on T2 wellbeing scores without adjusting for the effect of socio-demographic covariates (with the exception of T1 wellbeing) on T2 wellbeing (see Fig. S1 and Table S5, Supplementary Materials for conceptual diagram) was performed.

Missing data in the analytical sample ranged from 45.94% (T2 mental wellbeing scores) to 0.75% (social media use); see Fig. S2, Supplementary Materials). Missing data on the LCA indicators (PACE items; ranging from 0.81 to 2.39% across the individual items), outcome variable (T2 mental wellbeing, 45.94% missing in the analytical sample), and covariates were accounted for using Full Information Maximum Likelihood (FIML) (Enders, 2010), which is preferable to listwise deletion as it makes use of all available data and reduces bias due to attrition. However, for completeness, a sensitivity analysis was performed whereby individuals with missing data on any of the covariates (*n* = 4116; 22.59%) were deleted list-wise (the default in MPlus) and the 3-step method on the covariate complete case sample (*n* = 14,108; see Table S6, Supplementary Materials) was repeated.

## Results

### Model Selection

**Fig. 2:**
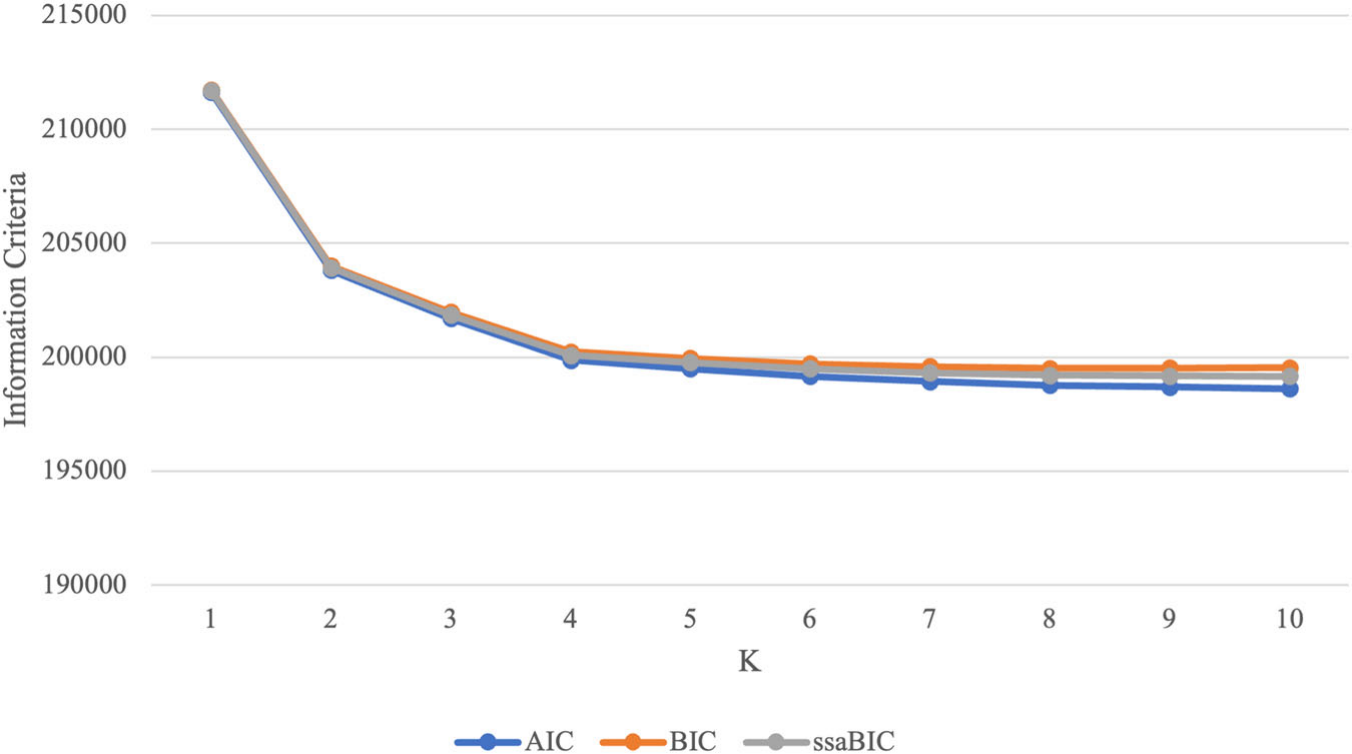
Information criteria for classes (K)

Fit indices, entropy, LMR-LRT *p* values, and class sizes for each latent class model can be found in Table 1. Increasing the number of classes in the model improved model fit, as indicated by a reduction in AIC, BIC and ssaBIC. However, a plot of the information criteria values (Fig. 2) suggested that the rate of improvement levelled off after the 4-class solution, and LMR-LRT p –values also indicated no significant improvement in model fit after that point. The 4-class model did not have any classes representing less than 10% of the sample, suggesting classes had not been over-extracted (Masyn, 2013). In addition, classes were distinct, having specific characteristic items and item response probabilities that differed across classes. In the split halves analysis, differences in PACE item endorsement rates across the two sample halves were negligible, suggesting that classes were reliably identified (average absolute differences in the probability of endorsing a given PACE item between the two halves ranged between 0.02 and 0.1; see Supplementary File).

**Table 1:** Fit statistics, classification indices and class sizes

K	LL	AIC	BIC	ssaBIC	Entropy	LMR LRT *p* value	Class size (proportion based on model estimate)
1	−105807.74	211637.5	211723.4	211688.4	NA	NA	1
2	−101889.65	203825.3	204004.9	203931.8	0.56	<0.001	0.52; 0.48
3	−100814.43	201698.9	201972.2	201861	0.59	<0.001	0.47; 0.27; 0.26
**4**	−**99889.97**	**199873.9**	**200241**	**200091.7**	**0.6**	**<0.001**	**0.29**; **0.19**; **0.40**; **0.12**
5	−99685.49	199489	199949.8	199762.3	0.58	0.048	0.10; 0.20; 0.30; 0.23; 0.16
6	−99509.01	199160	199714.6	199488.9	0.57	0.148	0.02; 0.13; 0.23; 0.21; 0.24; 0.16
7	−99384.63	198935.3	199583.5	199319.8	0.59	0.229	0.02; 0.23; 0.13; 0.20; 0.02; 0.25; 0.14
8	−99291.55	198773.1	199515.1	199213.2	0.56	0.373	0.21; 0.02; 0.15; 0.14; 0.19; 0.02; 0.14; 0.13
9	−99237.43	198688.9	199524.6	199184.6	0.56	0.519	0.15; 0.19; 0.08; 0.02; 0.12; 0.09; 0.17; 0.02; 0.17
10	−99187.26	198612.5	199542	199163.8	0.61	0.264	0.06; 0.01; 0.18; 0.15; 0.01; 0.09; 0.13; 0.07; 0.20; 0.09

Bold = final 4 class solution

*AIC* Akaike Information Criterion, *BIC* Bayesian Information Criterion, *K* number of classes, *LL* log-likelihood, *LMR-LRT* Lo–Mendell–Rubin Likelihood Ratio Test, *p* = *p* value, *ssaBIC* sample size adjusted BIC

Entropy is used as an indicator of overall classification quality (Masyn, 2013). For the 4-class model, entropy was 0.6 (see Table 1). Though entropy values closer to 1 indicate better classification quality, there is no agreed upon cut-off value, but over 0.8 is seen as preferable (Weller et al., 2020), with lower entropy values indicating ‘fuzzy’ classification^4^. However, although it is important to report entropy, it is not to be used for model selection (Masyn, 2013; Sinha, 2021). Further, entropy values ≥ 0.6 indicate sufficient class separation for 3-step methods (Asparouhov & Muthén, 2014). Accordingly, a 4-class model that included all PACE indicators was selected to incorporate the covariates and distal outcomes, and the appropriate three step covariate analysis was used to account for classification error when investigating relations with covariates and the outcome variable.

**Fig. 3:**
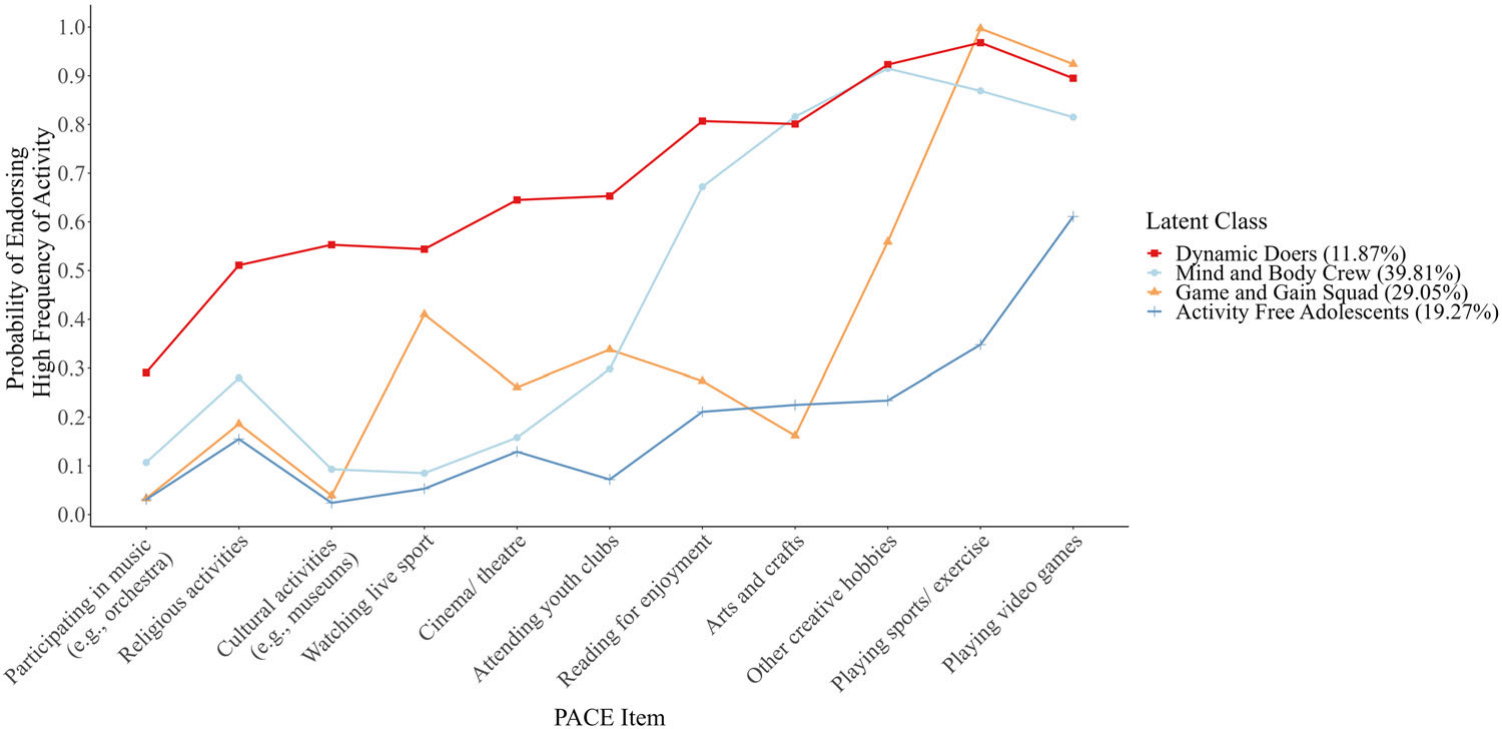
Class-specific item probability plot for 4 class model. PACE items are ordered according to endorsement frequency in the Dynamic Doers class, from the least to most endorsed item, so that variability between classes can be tracked

The four classes were named according to the patterns of responses to the frequency of taking part in various PACE activities (see Fig. 3). Class names were selected from a range of options provided by Chat GPT^5^. The classes were: (1) the Dynamic Doers, who had high probabilities of endorsement for a wide range of PACE items (11.87%); (2) the Mind and Body Crew, characterized by high probabilities of taking part in reading for enjoyment, arts and crafts, playing sports, doing exercise or other physical activities, playing games on a computer or games console, and participating in other creative activities (39.81%); (3) the Game and Gain Squad, who were highly likely to play sports, do exercise or other physical activities, and play games on a computer or games console (29.05%); and, (4) the Activity Free Adolescents, characterized by low probabilities of endorsing all PACE items (19.27%) (see Fig. 3).

### Predictors of Class Membership

**Fig. 4:**
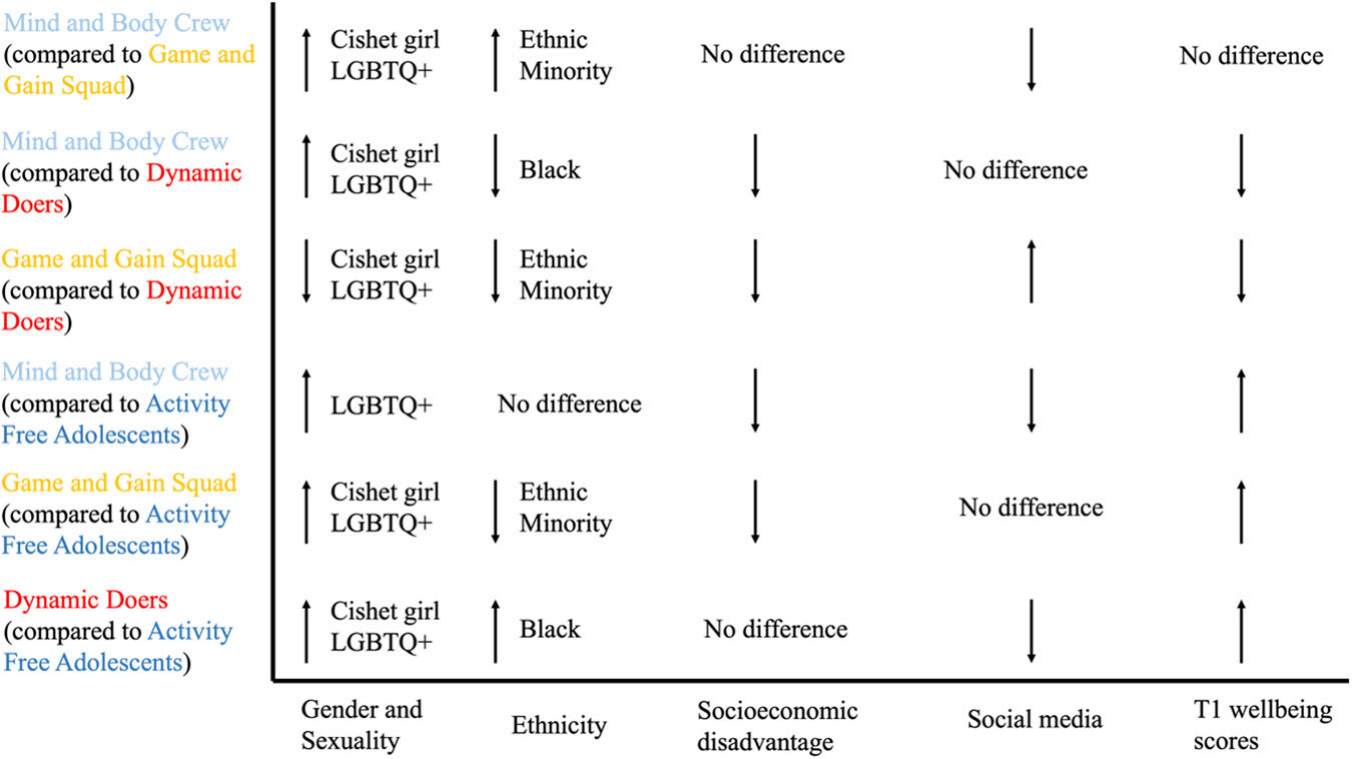
Visual representation of the Relationship Between Socio-Demographic Characteristics and PACE Latent Class Membership. No difference = Odds ratio was not statistically significant; ↓ = lower odds compared to reference group;↑ = higher odds compared to reference group

Compared to the Activity Free Adolescents, those in the Dynamic Doers class were less likely to be cisgender heterosexual girls or LGBTQ+, more likely to be Black, spent less time on social media, and had higher T1 wellbeing scores. Those in the Game and Gain Squad were less likely to be cisgender heterosexual girls, or LGBTQ+, to be from an ethnic minority group, or to be subject to socioeconomic deprivation, and had higher T1 wellbeing scores. Finally, those in the Mind and Body Crew were more likely to be LGBTQ+, less likely to be subject to socioeconomic deprivation, spent less time on social media, and had higher T1 wellbeing scores than those in the Activity Free Adolescents (see Table 2 and Fig. 4).

**Table 2:** Odds Ratios and 95% Confidence Intervals Showing the Relationship Between Socio-Demographic Characteristics and PACE Latent Class Membership

Covariate	Dynamic Doers	Game and Gain Squad	Mind and Body Crew	Activity Free Adolescents
Activity Free Adolescents as reference class				
Cisgender heterosexual girls	0.4 [0.31; 0.51]*	0.11 [0.09; 0.14]*	1.09 [0.88; 1.35]	–
LGBTQ+	0.64 [0.51; 0.81]*	0.13 [0.1; 0.16]*	1.44 [1.17; 1.77]*	–
Asian	1.01 [0.77; 1.34]	0.44 [0.34; 0.57]*	0.96 [0.79; 1.17]	–
Black	1.62 [1.18; 2.21]*	0.53 [0.39; 0.72]*	0.76 [0.58; 1]	–
Mixed	1.16 [0.77; 1.75]	0.74 [0.55; 0.99]*^a^	1.28 [0.98; 1.67]	–
Other (incl. Chinese)	1.09 [0.72; 1.63]	0.27 [0.16; 0.43]*	1.29 [0.89; 1.87]	–
Disadvantaged	0.94 [0.75; 1.19]	0.58 [0.47; 0.71]*	0.71 [0.6; 0.84]*	–
Social media use	0.85 [0.81; 0.88]*	1 [0.96; 1.04]	0.83 [0.8; 0.85]*	–
Wellbeing (Time 1)	1.19 [1.16; 1.21]*	1.1 [1.08; 1.12]*	1.09 [1.07; 1.1]*	–
Dynamic Doers as reference class				
Cisgender heterosexual girls	–	0.29 [0.23; 0.36]*	2.74 [2.16; 3.48]*	–
LGBTQ+	–	0.2 [0.16; 0.25]*	2.24 [1.80; 2.80]*	–
Asian	–	0.43 [0.33; 0.57]*	0.95 [0.75; 1.19]	–
Black	–	0.33 [0.25; 0.43]*	0.47 [0.35; 0.63]*	–
Mixed	–	0.64 [0.42; 0.97]*	1.1 [0.74; 1.64]	–
Other (incl. Chinese)	–	0.24 [0.14; 0.42]*	1.19 [0.78; 1.81]	–
Disadvantaged	–	0.61 [0.48; 0.78]*	0.75 [0.58; 0.98]*^a^	–
Social media use	–	1.18 [1.14; 1.23]*	0.97 [0.94; 1]	–
Wellbeing (Time 1)	–	0.93 [0.91; 0.95]*	0.92 [0.9; 0.93]*	–
Game and Gain Squad as reference class				
Cisgender heterosexual girls	–	–	9.62 [7.97; 11.61]*	–
LGBTQ+	–	–	11.29 [9.05; 14.08]*	–
Asian	–	–	2.18 [1.71; 2.79]*	–
Black	–	–	1.43 [1.05; 1.95]*	–
Mixed	–	–	1.72 [1.27; 2.34]*	–
Other (incl. Chinese)	–	–	4.86 [3.15; 7.51]*	–
Disadvantaged	–	–	1.24 [0.97; 1.58]	–
Social media use	–	–	0.82 [0.8; 0.85]*	–
Wellbeing (Time 1)	–	–	0.99 [0.97; 1]	–

*indicates statistically significant ORs (95% CIs do not cross 1)

^a^Cases where discrepancies were observed between FIML and complete case analyses

Compared to the Dynamic Doers, those in the Game and Gain Squad were less likely to be cisgender heterosexual girls or LGBTQ+, less likely to be from an ethnic minority group, or to be subject to socio-economic deprivation, spent more time on social media, and had lower T1 wellbeing scores. In contrast, those in the Mind and Body Crew were more likely to be cisgender heterosexual girls or LGBTQ + , less likely to be Black or to be subject to socioeconomic deprivation, and had lower T1 wellbeing scores (see Table 2 and Fig. 4).

Finally, compared to those in the Game and Gain Squad, those in the Mind and Body Crew were more likely to be cisgender heterosexual girls or LGBTQ + , or to be from an ethnic minority background, and spent less time on social media (see Table 2 and Fig. 4).

Sensitivity analyses using complete cases (see Table S6, Supplementary Materials) were generally consistent with the main FIML analyses (see Table 2 and Fig. 4), save for a small number of discrepancies. These pertained mainly to ethnic group, where findings in the FIML analyses were statistically significant, but not in the complete case analysis, or vice versa. These discrepancies are noted in Table 2.

### PACE Classification and Later Mental Wellbeing

**Fig. 5:**
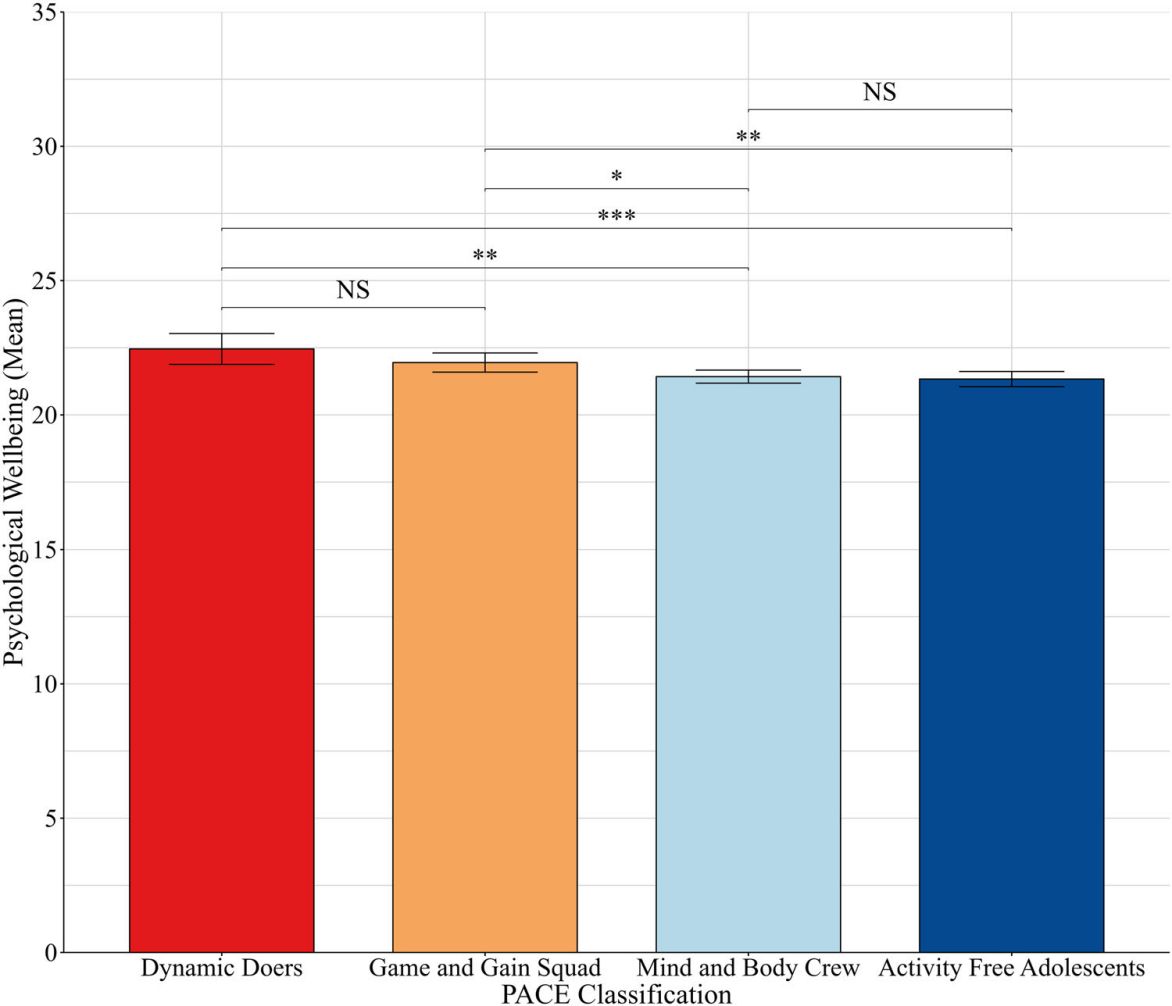
Error bar chart showing mean T2 mental wellbeing scores in each PACE class. Error bars indicate 95% confidence intervals. NS not significant; *p < 0.05; **p < 0.01; ***<0.001

Adolescents in the Dynamic Doers class had the highest mental wellbeing scores at T2 (mean 22.46), and those in the Activity Free Adolescents had the lowest scores (mean = 21.34) (see Fig. 5). T2 mental wellbeing was not significantly different between adolescents in the Dynamic Doers class and the Game and Gain Squad (d = 0.12, *p* = 0.124), or between those in the Mind and Body Crew and Activity Free Adolescents (d = 0.02, *p* = 0.555). However, adolescents in the Dynamic Doers class had significantly higher T2 mental wellbeing scores than adolescents in the Activity Free Adolescents (d = 0.26, *p* = <0.001), and adolescents in the Mind and Body Crew (mean = 21.43; d = 0.24, *p* = 0.002). Adolescents in the Game and Gain Squad also had higher T2 wellbeing scores (mean = 21.95) than those in the Mind and Body Crew (d = 0.12, *p* = 0.016) and the Activity Free Adolescents (d = 0.14, *p* = 0.009). These findings were broadly consistent with the sensitivity analysis on a complete case sample. The only exception was the comparison between the Dynamic Doers and Mind and Body Crew, which was not statistically significant in the complete case analysis (see Supplementary File Section 4).

## Discussion

Participation in arts, culture, and entertainment activities may promote adolescent wellbeing. However, little is known about how such activities cluster together, and previous research has predominantly used variable-focused approaches, small samples, and cross-sectional designs, has focused on single activities, and/or has not considered the influence of socio-demographic factors on participation. The current study was designed to address these limitations. It was driven by three research aims: to establish patterns (latent classes) of participation in arts, culture, and entertainment activities among adolescents in Greater Manchester, England (Research Question 1); to assess associations between these patterns and a range of covariates (e.g., social media use, ethnicity, socioeconomic disadvantage) (Research Question 2), and, to determine whether patterns of participation in arts, culture, and entertainment predicted later mental wellbeing (Research Question 3).

### Patterns of Participation in Arts, Culture, and Entertainment Activities (Research Question 1)

Four distinct patterns of participation in arts, culture, and entertainment activities were identified, providing insight into the differing ways adolescents use their leisure time, and addressing an important evidence gap about how different activities cluster together (Fancourt et al., 2021). Findings indicated that patterns of participation in arts, culture, and entertainment engagement vary across different groups of young people. For example, one class of adolescents (the Game and Gain Squad) spend a lot of their leisure time playing video games, sports, or doing exercise, at the expense of other activities. By contrast, another class of young people (the Mind and Body Crew) tend to spend time (albeit less than the Game and Gain Squad) undertaking these activities, and also read for pleasure, and engage in arts, crafts and other creative hobbies. Although both classes are characterized by activities which may be considered productive and goal-oriented, they are distinguished by the fact that the Mind and Body Crew participate in activities that offer creative/imaginative outlets, while the Game and Gain Squad focus their time specifically on activities which likely offer a competitive element.

A further group of adolescents (the Dynamic Doers) report engaging in a wide range of participation in arts, culture, and entertainment activities, akin to the *omnivore* class noted in the LCA of adult cultural, arts, heritage and sports activities (Walker et al., 2022). Working from a Bourdieusian perspective, the authors of that study hypothesized that wide-ranging consumption of arts, culture and entertainment reflects significant economic and social capital and advantage; however, our analysis did not support this proposition (see *Socio-demographic predictors of class membership* below). Another interesting distinction is the size of these analogous classes: while the Dynamic Doers represented nearly 12% of our sample, fewer than 5% of the adult study sample were classified as omnivores (Walker et al., 2022). Collectively, these contrasting findings are indicative of potentially important shifts in the drivers and prevalence of participation in arts, culture, and entertainment activity patterns between adolescence and adulthood (i.e., it may be the case that social and economic advantage becomes more central to wide-ranging participation in arts, culture, and entertainment engagement in adulthood, when leisure time reduces).

It is worth noting that even among the Dynamic Doers class, frequency of engagement in some activities remained relatively low, despite being higher than in other classes (e.g., watching live sport, playing in an orchestra/ a musical instrument, attending museums or art galleries, religious activities, or going to the cinema/theater). It is possible that the COVID-19 pandemic played a role in this, as the T1 participation in arts, culture, and entertainment data used in our analyses were collected between September and December 2021. In England, non-essential businesses such as museums and libraries reopened in April 2021; and cinemas reopened and large audiences at sporting events were once again permitted in May 2021 (Institute for Government Analysis, 2021). The low levels of engagement in these activities, even among the Dynamic Doers, may reflect the slow return to normality (ONS, 2022) and possible associated heightened feelings of anxiety following the multiple periods of lockdown that began in 2020 (Smithson, 2021). Indeed, a recent report noted that engagement in such activities is still below pre-pandemic levels for 16–19-year-olds (The Audience Agency, 2023). However, given the much higher levels of engagement in other activities that were similarly impacted by lockdown, such as sports participation, it may be that adolescents were prioritizing some forms of participation in arts, culture, and entertainment activities over others at T1, perhaps motivated by physical health goals following a lengthy sedentary period. An alternative explanation is that these kinds of activities are simply not favored by 12–13-year-olds, though this proposition is not supported by other research. For example, one study found that a third of 10–18 year-olds engaged in choirs, orchestras, theater and dancing, and this did not differ by age (Auhuber et al., 2019).

A final class of adolescents reported low levels of engagement in all activities (the Activity Free Adolescents), akin to the *disengaged* class in the aforementioned research on adult participation in arts, culture, and entertainment (Walker et al., 2022). However, even among this class, it is noteworthy that there was still a relatively high (0.6) probability of endorsement of frequently playing videogames (compared to probabilities ranging from 0.02 to 0.3 for the remaining activities). This is perhaps unsurprising, given the surge in gaming in recent years, particularly among adolescents, which are at an all-time high (for example, 91% of young people aged 16–24 reported engagement with some form of gaming in 2019; GWI, 2020). More broadly, the global prevalence of gaming is thought to be around 2.7 billion (GWI, 2021). Further, when access to other arts, culture, and entertainment activities was restricted during the COVID-19 lockdown periods, many people may have turned to gaming to fulfil entertainment and socializing needs (GWI, 2021). This type of activity notwithstanding, our findings show that close to 1 in 5 young people report minimal participation in arts, culture, and entertainment activities, which appears to increase to 1 in 4 by adulthood (Walker et al., 2022).

### Socio-Demographic Correlates of Participation in Arts, Culture, and Entertainment Class Membership (Research Question 2)

Belonging to classes that were characterized by a wider range of activities was associated with lower odds of social media use. For example, the Dynamic Doers and Mind and Body Crew spent significantly less time using social media than the Activity Free Adolescents and Game and Gain Squad. This pattern of findings aligns with previous research, which suggests that increased screen time (including social media use) displaces other activities (Auhuber et al., 2019). However, other research also suggests that high levels of both physical activity and screen time (defined as watching TV, playing video games, and smartphone use) can coincide with one another (Ferrar et al., 2013; Taverno Ross et al., 2016), particularly among boys. The fact that the Game and Gain Squad spent significantly more time using social media and were more likely to be boys than the Dynamic Doers and the Mind and Body Crew aligns with such findings (keeping in mind that adolescents’ use of social media is overwhelmingly through their smartphones; Vogels et al., 2022).

By contrast, findings regarding socio-economic disadvantage were less intuitive and were inconsistent with prior theory and evidence. Socio-economic disadvantage was not a significant predictor of Dynamic Doers class membership compared to the Activity Free Adolescents, yet those in the Mind and Body Crew and Game and Gain Squad were significantly less likely to be socioeconomically disadvantaged than other classes, including Dynamic Doers. As noted above, this diverges from the Bourdieusian analysis focusing on adults (Walker et al., 2022), in addition to research showing that engagement in arts, culture, and entertainment activities is higher among those from more affluent areas among adults (Mak et al., 2020), and young people outside of school (Mak & Fancourt, 2021). Furthermore, the cost of such activities has been cited as a barrier to participation (ART31, 2018). One may therefore have anticipated socioeconomic disadvantage to clearly and consistently distinguish our classes in expected ways. It is possible that these surprising findings reflect the derivation of the measure of socioeconomic disadvantage used in the current study, which required adolescents to be both eligible for free school meals *and* resident in the lowest IMD quintile. While this addressed the issue of inclusivity (i.e., the c.11% classified as disadvantaged were subject to both familial *and* neighborhood socio-economic deprivation), the binary classification approach used meant the measure was insensitive to gradients of socio-economic (dis)advantage (i.e., socio-economic differences between our classes could be more evident among those in the remaining c.89% of our sample, which was inevitably heterogenous).

It should also be noted that socio-economic (dis)advantage is a multi-faceted concept, and other distinct components not captured in the measure, such as parental education, may be important in distinguishing classes. For example, participation in musical activities such as choirs or orchestras, or attendance at museums or the theater outside of school may be influenced by parental exposure (both in childhood and adulthood) or interest in such activities (Mak et al., 2020; Mak & Fancourt, 2021). Parental exposure to these activities may in turn by influenced by parent education (i.e., parent education as a predictor of engagement in the arts and cultural activities, with parents with higher levels of education being more likely to engage in such activities, as has been the case among adults; for example, Walker et al., 2022). Finally, it is also important to consider the potential influence of the cost-of-living crisis, resulting from which overall lower levels of participation in entertainment outside of the home have been reported (Torreggiani, 2022); it is possible that this is the case regardless of level of socioeconomic disadvantage.

Sexual orientation and gender identity also predicted participation in arts, culture, and entertainment class membership. Adolescents in the Mind and Body Crew were more likely to identify as cisgender heterosexual girls or LGBTQ+ compared to those in the Game and Gain Squad. Although high levels of playing videogames, sports and exercise is characteristic of both classes, those in the Mind and Body Crew additionally engage in more creative activities. These findings align with previous research indicating that girls are more likely to participate in sedentary activities (Leech et al., 2014), whereas boys are more likely to be physically active (Hallal et al., 2012). Those in the Mind and Body Crew are also more likely identify as cisgender heterosexual girls or LGBTQ+ than those in the Dynamic Doers class. This contrasts with other work, where girls were more likely to engage in playing music or being part of an orchestra and attending the theater or dancing (Auhuber et al., 2019). Dynamic Doers had the highest probability of attending the cinema or theater, and participating in musical activities, so one may have expected, in line with this previous work, that identifying as a cisgender heterosexual girl would predict membership to this class (compared to others). However, activities characteristic of the Mind and Body Crew (e.g., reading for pleasure, arts, crafts, and other creative hobbies) can be engaged in at home or alone, whereas the additional activities that are endorsed by the Dynamic Doers (for example, attending youth clubs or museums) involve community spaces and interacting with others. It is possible that adolescents who identify as cisgender heterosexual girls or LGBTQ+ perceive such community spaces and/or activities to be inaccessible or unsafe and so orient to more home-based activities. This proposition is offered partial support by a recent analysis of the #BeeWell dataset which indicated that trans and gender diverse adolescents (but not cisgender girls) felt significantly less safe in their local area than cisgender boys (Black, Humphrey, & Marquez, 2023.

The most clear and consistent pattern observed in relation to ethnicity was the reduced likelihood of Game and Gain Squad membership among adolescents from all minority ethnic groups, across all contrasts (i.e., compared to classification as Dynamic Doers, Activity Free Adolescents, and Mind and Body Crew). In addition, Black adolescents were significantly more likely to be in the Dynamic Doers class than in the Activity Free Adolescents. These findings appear indicative of an ‘all or nothing’ trend among adolescents from ethnic minority groups (particularly Black adolescents). That is, these adolescents are much more likely to either to engage in *wide-ranging* or *minimal* arts, culture, and entertainment activities than they are to engage selectively and specifically in video games, sports and exercise and/or arts, crafts and other creative hobbies. Ethnic minority adolescents who endorse such activities do so as part of a broader pattern of omnivorous arts, culture and entertainment consumption (i.e., Dynamic Doers), and those who do not typically do not endorse *any* arts, culture, and entertainment activities (i.e., Activity Free Adolescents). The latter finding resonates with statistics indicating that individuals from ethnic minority groups make up just 7% of adult audiences for the arts (Arts Council England, 2022), despite constituting nearly 20% of the population in England (HM Government, 2023). Cultural context is an important factor in determining participation and attendance rates, and individuals from ethnic minority backgrounds may be less likely to engage in certain arts, culture, and entertainment activities due to perceptions of cultural incongruence, discomfort, and/or a lack of representation (Arts Council England, 2016; Bridgwood et al., 2003). In support of this, adults from ethnic minority groups were significantly more likely to belong to the *disengaged* class than their White counterparts (Walker et al., 2022).

However, these factors do not explain the former finding, relating to wide-ranging arts, culture, and entertainment engagement. Ethnicity, by definition, refers to a shared cultural background, which can encompass religion, food, music, and traditions, among other things (Porta & Last, 2018). Adolescents from ethnic minority groups who tend towards more wide-ranging arts, culture, and entertainment engagement may do so because they spend their leisure time actively engaging in traditional or cultural events, such as community celebrations, parades, and festivals (Mak & Fancourt, 2021). In support of this proposition is the fact that the participation in arts, culture, and entertainment items ‘attend a religious service’ had a higher rate of endorsement among the Dynamic Doers than any other latent class in the current study. It is also possible that engagement in a range of arts, culture, and entertainment activities facilitates a sense of cultural/group belonging, and/or socialization, among ethnic minority group members (Arts Council England, 2016).

### Participation in Arts, Culture and Entertainment Class Membership and Later Wellbeing (Research Question 3)

Among young people, engagement in arts, culture and entertainment activities can bring a sense of group identity and social belonging, provide a space to interact with peers with similar interests, improve social skills, and provide a foundation upon which to build new relationships, all of which may produce wellbeing benefits (Bone & Fancourt, 2022; Rees, 2018). Indeed, links between engagement in arts and cultural activities and wellbeing are well established (albeit primarily with adult samples). For example, studies have shown engagement in reading for pleasure, creative activities and art activities improved mental wellbeing, both before the COVID-19 pandemic (Rees, 2018; Zarobe & Bungay, 2017), and more recently during the lockdown periods (Bone et al., 2023). Such activities have also been used as a therapeutic approach for those with both physical and mental health conditions (Jensen et al., 2016). Similarly, physical activity is consistently identified as a contributing factor to positive psychological wellbeing (save for a minority of studies, e.g., Bell et al., 2019). This relationship has been found to be reciprocal (Marsigliante et al., 2023) and amenable to intervention (e.g., a school-based physical activity intervention was found to improve psychological wellbeing among children and young people; Marsigliante et al., 2023). Finally, research pertaining to the impact of gaming has historically focused on negative otucomes such as addiction and aggression, however, there is now evidence to suggest that playing videogames can be beneficial to wellbeing and psychological functioning (Adachi & Willoughby, 2017), or indeed have little to no effect on wellbeing, as has been reported in one study (Vuorre et al., 2022).

The current study addresses a range of previously noted methodological limitations in studies investigating these links (Bone & Fancourt, 2022), and our findings add rigorous longitudinal evidence pertaining to adolescence. After controlling for prior levels of wellbeing and other covariates, a clear pattern was evident in which engagement in either wide-ranging (Dynamic Doers) or selective (Game and Gain Squad) arts, culture, and entertainment activities resulted in improved wellbeing, compared to minimal engagement (Activity Free Adolescents). Notably, the magnitude of this association was stronger in the former contrast (d = 0.26) compared to the latter (d = 0.14). Furthermore, engagement in wide-ranging activities (Dynamic Doers) resulted in improved wellbeing compared to one of the selective classes (Mind and Body Crew) (d = 0.24). Collectively, these analyses support the proposition that relative levels of time spent on a variety of arts, culture, and entertainment activities may be crucial, as opposed to simply the time spent on specific activities (Bone & Fancourt, 2022).

Average wellbeing scores for Dynamic Doers did not differ from those in the Game and Gain Squad (who engage specifically in regular sports, exercise, and video games). It might be tempting to conclude, therefore, that among the range of arts, culture, and entertainment activities, physical activity may be particularly beneficial for wellbeing. This could be due to relief from feelings of stress and anxiety (via improved functioning of the hypothalamus-pituitary-adrenal axis; Mahindru et al., 2023), decreases in sedentary time, and/or increased feelings of satisfaction about one’s health (Rees, 2018). However, in the current study, average wellbeing scores for Dynamic Doers were significantly higher than those in the Mind and Body Crew (whose pattern of participation also includes regular sports/exercise). Similarly, average wellbeing scores for the Mind and Body Crew did not differ significantly from the Activity Free Adolescents. Thus, more frequent videogaming (a distinguishing feature between the Game and Gain Squad and Mind and Body Crew, with a probability of 0.92 compared to 0.82) may actually be a particularly ‘active ingredient’ among the range of participation in arts, culture, and entertainment activities. In support of this proposition, the positive effects of video gaming on adolescent wellbeing were noted in a recent review (Adachi & Willoughby, 2017), in which the authors used self-determination theory as an explanatory framework (i.e., video gaming is theorized to benefit wellbeing because it supports a sense of efficacy, personal agency, and social connectedness). Other work has suggested that playing videogames acts as an outlet for young people to release feelings of frustration and aggression, with reports of improved psychological states after a gaming session (Jones et al., 2014). The motivations behind engaging with videogames is also thought to be an important moderating factor for the effects on wellbeing (Halbrook et al., 2019).

The relationship between engaging in arts, culture, and entertainment activities and wellbeing is assumed to be reciprocal, with those experiencing higher levels of wellbeing more likely to subsequently engage in arts, culture, and entertainment activities (Bone & Fancourt, 2022; Rees, 2018). This is partially supported by the fact that concurrent wellbeing (measured at T1 alongside data on participation in arts, culture, and entertainment activities) predicted class membership. Dynamic Doers, Game and Gain Squad and Mind and Body Crew members all had significantly higher T1 wellbeing scores than Activity Free Adolescents, and Dynamic Doers had higher T1 wellbeing scores than those in the Mind and Body Crew and the Game and Gain Squad.

### Implications, Limitations and Future Directions

The current study provides robust evidence of the connection between participation in arts, culture, and entertainment activities and improved wellbeing. In the context of significant societal concern about adolescent wellbeing (Mei et al., 2020), a key implication of the current study is that further engagement in arts, culture, and entertainment activities may yield meaningful benefits in this regard. Of particular note are the c. 1 in 5 disengaged young people (the Activity Free Adolescents), and the evidence of socio-demographic inequalities in participation. The analytical sample closely mirrored the 11–16 population of adolescents in Greater Manchester and nationally (albeit with some differences in ethnic composition), giving confidence that the findings are generalizable to young people across England. However, findings may not be generalizable in a more global context. Nonetheless, investment in the prioritization and promotion of provision relating to arts, culture and entertainment should therefore be prioritized. This could be through both reinvigoration of arts and cultural provision in schools (i.e., to address the aforementioned decline in the number of arts-specialist teachers, and the time spent teaching arts-based subjects) and as part of universal personalized care (i.e., through social prescribing initiatives). Particular attention should be paid to ways in which a full range of activities can be made appealing and accessible to all young people. Furthermore, given the findings pertaining to the distinct benefits of video gaming, there is perhaps work to be done to change the public perception of this form of entertainment. Far from being a frivolous use of leisure time that could in fact lead to negative outcomes (the lens through which video gaming has been, and continues to be viewed, by many; Adachi & Willoughby, 2017), the findings of this study indicate that more time spent video gaming may in fact be *time well spent*.

In considering the above-noted implications, however, it is important to be cognizant of a number of limitations of the current study. First, though a relatively large and wide-ranging set of participation in arts, culture, and entertainment items was used, these were by no means all encompassing. Relatedly, when thinking specifically about adolescents from ethnic minority groups, their cultural and social diversity means that it is possible that there are other leisure time activities that are not covered in the participation in arts, culture, and entertainment item set (e.g., food festivals, which may make up a large part of their cultural experience; Arts Council England, 2016). Second, as previously mentioned, information about engagement in participation in arts, culture, and entertainment activities was collected in Autumn 2021, and it is possible that the Covid-19 pandemic and resulting restrictions also influenced reported engagement levels. Third, the nature of the LCA made it necessary to truncate participation in arts, culture, and entertainment item responses (i.e., “Most Days”, “At Least Once a Week”, and “At Least Once a Month” responses classified as high engagement). As a result, the analysis was insensitive to the more granular dose-response relationship between engaging in the arts and culture and later wellbeing that has been noted elsewhere in the literature (Bone & Fancourt, 2022). For example, more frequent engagement in participation in arts, culture, and entertainment activities has been found to be associated with larger increases in wellbeing: daily engagement was associated with greater increases than weekly engagement, which was associated with greater increases than monthly engagement (Bone et al., 2023). Alternatively, it has been suggested that continuous engagement with participation in such activities is necessary for sustained effects on later wellbeing, otherwise benefits may atrophy (Bone & Fancourt, 2022). It is possible that the levels of engagement in some activities reported at T1 may have changed in the period leading to T2, affecting the magnitude of impact on wellbeing. Future research with shorter lags and more frequent data points, analyzed using methods that enable researchers to pinpoint the nature and magnitude of reciprocal, within-person associations between participation in arts, culture, and entertainment and wellbeing (e.g., random intercept cross-lagged panel models; Hamaker et al., 2015), is therefore recommended.

A fourth limitation of the current study is that, while the analysis was able to robustly identify patterns of participation in arts, culture, and entertainment activity and examine associations with socio-demographic characteristics and wellbeing, it did not consider potential moderators and mechanisms that would help to explain these associations. For example, young people’s perceived agency (i.e., the extent to which they feel able to choose how to spend their free time, as opposed to this being dictated to them by parents) to engage in leisure activities of their choosing may be an important factor in the effects of participation in arts, culture, and entertainment engagement on later wellbeing, with those who perceive greater autonomy experiencing better wellbeing (Kuykendall et al., 2018). Similarly, future research could examine if the wellbeing benefits of participation in arts, culture, and entertainment observed here result from more effective emotional regulation and coping, enhanced vitality, and/or increased development of self-identity, self-acceptance, and social connectedness. For example, it is plausible to assume that young people who engage in a broad range of activities (such as the Dynamic Doers class established in the current study) would have more social connections than those who do not engage in a broad range of activities (the Activity Free Adolescents), presenting another potential moderator of the influence of said activities on wellbeing. Indeed, social connectedness may act as a protective factor against mental ill health among adults in the general population (Wickramaratne et al., 2022), and is thought to be critical to the foundations for healthy development and positive wellbeing in adolescence (Blum et al., 2022).

## Conclusion

Participation in arts, culture, and entertainment activities is thought to promote adolescent wellbeing. However, little is known from the existing literature about how such activities cluster together, and previous research has used small samples, cross-sectional designs, focused on single activities, and/or has not considered the influence of socio-demographic factors on participation. The current study sought to address these research gaps, by establishing patterns (latent classes) of adolescent participation in arts, culture, and entertainment activities; assessing associations between these patterns and a range of covariates (e.g., social media use, ethnicity, socioeconomic disadvantage); and, determining whether latent class membership predicted later mental wellbeing. In conclusion, four distinct classes of adolescent participation in arts, culture, and entertainment were evident: Dynamic Doers (those who regularly participated in a wide range of participation in arts, culture, and entertainment activities); the Game and Gain Squad (those who spent most of their free time playing video games, sports or exercising); the Mind and Body Crew (those who frequently engaged in reading for pleasure, arts and crafts, and other creative hobbies, in addition to playing video games, sports or exercising); and, the Activity Free Adolescents (those who reported low levels of participation in all activities). Membership of said classes was associated with a range of socio-demographic characteristics. Finally, and critically, participation in arts, culture, and entertainment activity patterns reflecting wide-ranging (Dynamic Doers) and selective (Game and Gain Squad) consumption were found to yield meaningful wellbeing benefits. Accordingly, investment in the prioritization and promotion of provision relating to arts, culture and entertainment for young people is warranted.

### Supplementary information

Supplementary Information

## Data Availability

RStudio code was used to prepare the analysis dataset, Mplus and Mplus Automation in RStudio was used for the analysis and data visualization. All analysis syntax is available on the Open Science Framework: 10.17605/OSF.IO/2JTPD.

## Footnotes

1 The #BeeWell study includes short (c.25% of the length of the full survey, with visual augments to facilitate understanding of response format) and symbol (c.10% of the length of the fully survey, items presented using Widgit symbols) survey versions that are available as options for young people in non-mainstream schools. These alternative versions were incompatible with the current study due to lack of coverage of key variables (e.g., mental wellbeing).

2 Even though these analyses were not focused on higher level data (i.e., explanatory variables at the school level), it is still important to account for the clustered nature of the underpinning dataset. This includes excising clusters with very small numbers of respondents, and use of the Type=Complex function in Mplus, in order to ensure that standard errors are estimated correctly.

3 Note that the ‘Any Other Ethnic Group’ and ‘Chinese’ categories were combined due to low Ns in both of these groups in the #BeeWell data, which would likely result in convergence issues if considered separately (Thornton & Humphrey, 2023).

4 It has been suggested that item quality is important for entropy, i.e., including items that distinguish classes from one another (Sinha et al., 2021). The class enumeration process was therefore repeated, removing the PACE indicator “Sing in a choir or play in a band or orchestra”, given the almost universally low endorsement of this activity. However, entropy for this 4-class model was 0.595. Further, examination of odds-ratios revealed that each PACE indicator separated at least two classes well, and classes were homogenous, with item probabilities for the “Sing in a choir or play in a band or orchestra” indicator being >0.7 or <0.3 in all four estimated classes (see Supplementary File, Section 5).

5 The following was entered into Chat GPT: Please can you help me come up with some catchy names that would be appropriate for latent classes, for groups characterized by the following: “1. High endorses of all activities; 2. High endorsers of playing video games and sports/exercise; 3. High endorsers of reading, crafts, video games, sports/exercise; 4. Low endorsers of everything”; along with the list of PACE items. Resulting suggestions were discussed, and those which included adjectives implying reasons for engagement in these activities (e.g., “The Relaxed Rejectors” for the Activity Free Adolescents, or “The Active Introverts” for the Mind and Body Crew) were avoided, as these included inferences beyond what the study dataset was able to show.
