# Supplementary material for: Do Patterns of Adolescent Participation in Arts, Culture and Entertainment Activities Predict Later Wellbeing? A Latent Class Analysis
Source: J Youth Adolesc. 2024 Mar 11;53(6):1396–414. doi: 10.1007/s10964-024-01950-7 (PMC11045570; doi:10.1007/s10964-024-01950-7)
Supplement: Supplementary file 1 — Supplementary Information [file 10964_2024_1950_MOESM1_ESM.docx]

**Do patterns of adolescent participation in arts, culture and entertainment activities predict later wellbeing? A latent class analysis: Supplementary Materials**

Table of Contents

[Section 1: Comparing analytical sample with Greater Manchester and National Populations 2](#_Toc146287101)

[Section 2: Split Halves Analyses 3](#_Toc146287102)

[Section 3: Relationship between PACE class membership and T2 Wellbeing (unadjusted) 5](#_Toc146287103)

[Section 4: Missing Data and complete case analyses for relation between PACE class membership and T2 Wellbeing 6](#_Toc146287104)

[Section 5: Class separation and item homogeneity 9](#_Toc146287105)

# **Section 1: Comparing analytical sample with Greater Manchester and National Populations**

Table S1: Comparison of analytic sample with Greater Manchester (GM) and National Population Statistics

|  | % | | | | |
| --- | --- | --- | --- | --- | --- |
|  | Sex | FSM | EAL | SEND | Ethnicity |
| Analytical sample | F = 50.54  M=49.38  NA = 0.08 | N = 72.21  Y = 25.49  NA = 2.30 | N = 78.07  Y = 20.62  NA = 1.32 | N = 83.59  Y = 14.90  NA = 1.51 | AOEG = 2.23  Asian = 17.15  Black = 4.87  Chinese = 0.95  Mixed = 5.75  Unclassified = 2.07  White = 65.10  NA = 1.88 |
| GM population^a^ | F = 48.60  M = 51.40 | Data not available^c^ | N = 78.46  Y = 21.10 | N = 85.81  Y = 14.19 | AOEG = 2.77  Asian = 17.1  Black = 6.55  Chinese = 0.65  Mixed = 6.32  Unclassified = 1.28  White = 65.4 |
| National statistics^b^ | F = 50  M = 50 | Data not available^c^ | N **= 80.1**  Y = 19.5 | N = 83.4  Y = 16.6 | AOEG: 2.2  Asian = 11.7  Black = 5.8  Chinese = 0.6  Mixed = 6.6  Unclassified = 1.6  White = 71.4 |

^a^ Greater Manchester (GM) population statistics: see Table S1 [here](https://gmbeewell.org/wp-content/uploads/2022/03/BeeWell-Inequalities-Evidence-Briefing.pdf)

^b^ National statistics: see [here](https://explore-education-statistics.service.gov.uk/find-statistics/school-pupils-and-their-characteristics/2021-22)

^c^ GM and national statistics only available for current FSM eligibility as opposed to EverFSM6 metric used in the current study

# **Section 2: Split Halves Analyses**

Table S2: Item response probabilities in the split half analysis: Split 1

| Item | Dynamic Doers | Game and Gain Squad | Mind and Body Crew | Activity Free Adolescents |
| --- | --- | --- | --- | --- |
| Cinema | 0.691 | 0.251 | 0.158 | 0.135 |
| Watching sport | 0.583 | 0.387 | 0.085 | 0.057 |
| Music | 0.299 | 0.029 | 0.103 | 0.03 |
| Reading | 0.794 | 0.268 | 0.671 | 0.204 |
| Clubs | 0.667 | 0.345 | 0.297 | 0.059 |
| Culture | 0.568 | 0.032 | 0.092 | 0.028 |
| Religion | 0.522 | 0.188 | 0.277 | 0.146 |
| Crafts | 0.795 | 0.157 | 0.816 | 0.224 |
| Gaming | 0.905 | 0.924 | 0.812 | 0.597 |
| Playing sports | 0.974 | 0.999 | 0.869 | 0.314 |
| Other | 0.931 | 0.539 | 0.906 | 0.22 |

Table S3: Item response probabilities in the split half analysis: Split 2

| Item | Dynamic Doers | Game and Gain Squad | Mind and Body Crew | Activity Free Adolescents |
| --- | --- | --- | --- | --- |
| Cinema | 0.588 | 0.269 | 0.157 | 0.123 |
| Watching sport | 0.498 | 0.43 | 0.082 | 0.049 |
| Music | 0.28 | 0.036 | 0.111 | 0.031 |
| Reading | 0.818 | 0.279 | 0.67 | 0.218 |
| Clubs | 0.637 | 0.327 | 0.297 | 0.082 |
| Culture | 0.524 | 0.046 | 0.093 | 0.019 |
| Religion | 0.495 | 0.184 | 0.281 | 0.164 |
| Crafts | 0.804 | 0.166 | 0.815 | 0.224 |
| Gaming | 0.885 | 0.922 | 0.818 | 0.621 |
| Playing sports | 0.962 | 0.994 | 0.866 | 0.372 |
| Other | 0.913 | 0.575 | 0.925 | 0.243 |

Table S4: Differences in item endorsement probabilities between Split 1 and Split 2

|  |  |  |  |  |
| --- | --- | --- | --- | --- |
| Item | Dynamic Doers | Game and Gain Squad | Mind and Body Crew | Activity Free Adolescents |
| Cinema | 0.103 | 0.018 | 0.001 | 0.012 |
| Watching sport | 0.085 | 0.043 | 0.003 | 0.008 |
| Music | 0.019 | 0.007 | 0.008 | 0.001 |
| Reading | 0.024 | 0.011 | 0.001 | 0.014 |
| Clubs | 0.03 | 0.018 | 0 | 0.023 |
| Culture | 0.044 | 0.014 | 0.001 | 0.009 |
| Religion | 0.027 | 0.004 | 0.004 | 0.018 |
| Crafts | 0.009 | 0.009 | 0.001 | 0 |
| Gaming | 0.02 | 0.002 | 0.006 | 0.024 |
| Playing sports | 0.012 | 0.005 | 0.003 | 0.058 |
| Other | 0.018 | 0.036 | 0.019 | 0.023 |
| Average absolute difference | 0.103 | 0.043 | 0.019 | 0.058 |

# **Section 3: Relationship between PACE class membership and T2 Wellbeing (unadjusted)**


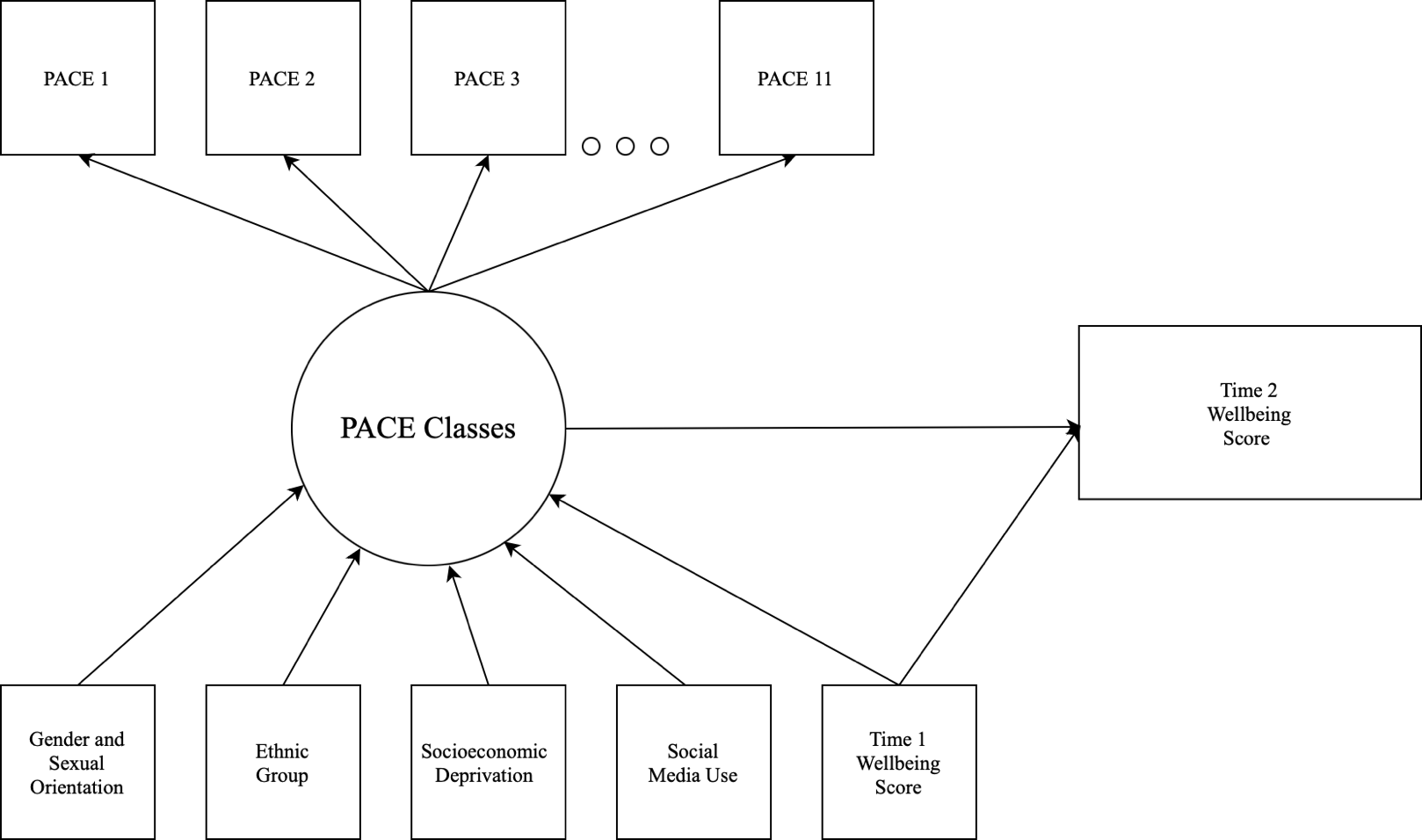
Figure S1: Conceptual diagram showing the unadjusted latent class model

Table S5: PACE Classification and Later Mental Wellbeing: Adjusted Model compared to Unadjusted model

|  | Effect Size (d), *p* value | |
| --- | --- | --- |
|  | Adjusted | Unadjusted |
| Dynamic Doers Class vs Game and Gain Squad | .12, p =.124 | .09, p =.262 |
| Dynamic Doers Class vs Mind and Body Crew | .24, p = .002 | .37, p <.001 |
| Dynamic Doers Class vs Activity Free Adolescents | .26, p <.001 | .43, p<.001 |
| Game and Gain Squad vs Mind and Body Crew | .12, p = .016 | .28, p<.001 |
| Game and Gain Squad vs Activity Free Adolescents | .14, p = .009 | .33, p<.001 |
| Mind and Body Crew vs Activity Free Adolescents | .02, p = .555 | .05, p = .136 |

# **Section 4: Missing Data and complete case analyses for relation between PACE class membership and T2 Wellbeing**

Figure S2: Proportion of missing data in analytical sample


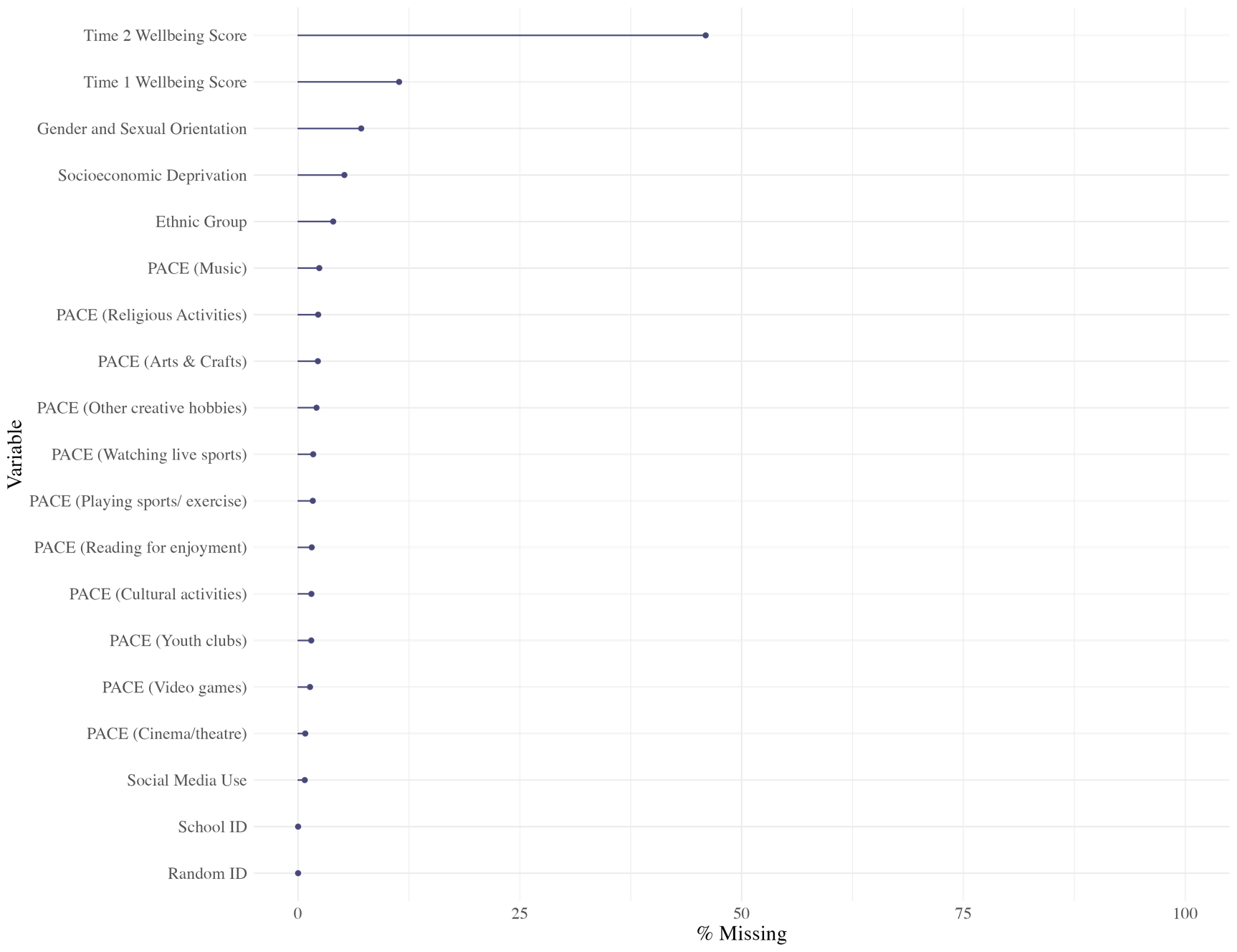


Table S6: Odds Ratios and 95% Confidence Intervals Showing the Relationship Between Class and Predictor Variables: Complete Case Analyses

| Covariate | Dynamic Doers Class | Game and Gain Squad | Mind and Body Crew | Activity Free Adolescents |
| --- | --- | --- | --- | --- |
| *Activity Free Adolescents as reference class* | |  |  |  |
| Cisgender heterosexual girls | 0.44 [0.33; 0.58]* | 0.11 [0.09; 0.14]* | 1.11 [0.88; 1.41] | - |
| LGBTQ+ | 0.67 [0.51; 0.88]* | 0.12 [0.1; 0.16]* | 1.51 [1.2; 1.9]* | - |
| Asian | 1.14 [0.86; 1.51] | 0.47 [0.36; 0.63]* | 1 [0.82; 1.22] | - |
| Black | 1.73 [1.19; 2.51]* | 0.47 [0.33; 0.66]* | 0.76 [0.56; 1.04] | - |
| Mixed | 1.17 [0.76; 1.81] | 0.75 [0.55; 1.01] ^a^ | 1.17 [0.89; 1.53] | - |
| Other (incl. Chinese) | 1.07 [0.7; 1.65] | 0.27 [0.16; 0.45]* | 1.37 [0.91; 2.05] | - |
| Disadvantaged | 0.86 [0.65; 1.14] | 0.57 [0.45; 0.73]* | 0.71 [0.59; 0.86]* | - |
| Social media use | 0.83 [0.79; 0.87]* | 0.98 [0.94; 1.03] | 0.81 [0.78; 0.84]* | - |
| Wellbeing (Time 1) | 1.19 [1.16; 1.22]* | 1.1 [1.08; 1.13]* | 1.08 [1.07; 1.1]* | - |
| *Dynamic Doers Class as reference class* | |  |  |  |
| Cisgender heterosexual girls | - | 0.26 [0.21; 0.33]* | 2.55 [1.97; 3.31]* | - |
| LGBTQ+ | - | 0.19 [0.14; 0.24]* | 2.25 [1.75; 2.89]* | - |
| Asian | - | 0.42 [0.31; 0.56]* | 0.88 [0.7; 1.12] | - |
| Black | - | 0.27 [0.19; 0.37]* | 0.44 [0.32; 0.6]* | - |
| Mixed | - | 0.64 [0.41; 0.99]* | 1 [0.65; 1.54] | - |
| Other (incl. Chinese) | - | 0.25 [0.15; 0.42]* | 1.28 [0.82; 1.99] | - |
| Disadvantaged | - | 0.66 [0.49; 0.9]* | 0.82 [0.61; 1.11] ^a^ | - |
| Social media use | - | 1.19 [1.14; 1.24]* | 0.98 [0.94; 1.01] | - |
| Wellbeing (Time 1) | - | 0.93 [0.91; 0.95]* | 0.91 [0.89; 0.93]* | - |
| *Game and Gain Squad as reference class* | |  |  |  |
| Cisgender heterosexual girls | - | - | 9.76 [7.93; 12.01]* | - |
| LGBTQ+ | - | - | 12.17 [9.55; 15.51]* | - |
| Asian | - | - | 2.11 [1.62; 2.76]* | - |
| Black | - | - | 1.63 [1.16; 2.31]* | - |
| Mixed | - | - | 1.56 [1.12; 2.17]* | - |
| Other (incl. Chinese) | - | - | 5.15 [3.2; 8.27]* | - |
| Disadvantaged | - | - | 1.25 [0.96; 1.62] | - |
| Social media use | - | - | 0.82 [0.79; 0.85]* | - |
| Wellbeing (Time 1) | - | - | 0.98 [0.97; 1] | - |

* indicates significant ORs (95% CIs do not cross 1)

^a^ Cases where discrepancies in statistical significance were observed between FIML and complete case analyses

Adolescents in the Dynamic Doers Class had the highest wellbeing scores at T2, and adolescents in the Activity Free Adolescents had the lowest wellbeing scores at T2. Time 2 wellbeing was not significantly different between adolescents in the Dynamic Doers Class and the Game and Gain Squad (d = .04, *p*=.647*)*, between adolescents in the Dynamic Doers Class and the Mind and Body Crew (d = .16, *p* = .053), or between adolescents in the Mind and Body Crew and Activity Free Adolescents (d =.04, *p* = .300). However, adolescents in the Dynamic Doers Class had significantly higher T2 wellbeing scores (mean = 23.31) than adolescents in the Activity Free Adolescents (mean = 19.98; d = .2, *p* = .010). Adolescents in the Game and Gain Squad had higher T2 wellbeing scores (mean = 22.56) than adolescents in the Mind and Body Crew (mean = 21.17; d = .12, *p=*.025) and those in the Activity Free Adolescents (d = .16, *p=*.007).

# **Section 5: Class separation and item homogeneity**

Table S7: Odds ratios showing separation between classes

| PACE item | Class 1 vs Class 2 | Class 1 vs Class 3 | Class 1 vs Class 4 | Class 2 vs Class 3 | Class 2 vs Class 4 | Class 3 vs Class 4 |
| --- | --- | --- | --- | --- | --- | --- |
| Cinema | 2.39 | 1.88 | **0.19** | 0.79 | **0.08** | **0.10** |
| Wating Sport | **12.43** | **7.51** | 0.58 | 0.6 | **0.05** | **0.08** |
| Music (e.g., orchestra) | 1.06 | 0.28 | **0.08** | 0.27 | **0.08** | 0.29 |
| Reading for Fun | 1.41 | **0.18** | **0.09** | **0.13** | **0.06** | 0.49 |
| Youth Clubs | **6.59** | 1.2 | 0.271 | **0.18** | **0.04** | 0.23 |
| Cultural activities (e.g., museums) | 1.67 | 0.4 | **0.03** | 0.24 | **0.02** | **0.08** |
| Religious activities | 1.25 | 0.59 | 0.22 | 0.47 | **0.18** | 0.37 |
| Arts and Crafts | 0.67 | 0.04 | **0.05** | **0.07** | **0.07** | 1.1 |
| Video games | **7.72** | 2.75 | 1.42 | 0.36 | **0.18** | 0.52 |
| Playing sports/exercise | **592.90** | **47.76** | **10.54** | **0.08** | **0.02** | 0.22 |
| Other creative hobbies | 4.15 | **0.12** | **0.11** | **0.03** | **0.03** | 0.90 |

*Odds ratios >5 or <0.2 are bolded to indicate a high degree of class separation*

*Class 1 = Game and Gain Squad; Class 2 = Activity Free Adolescents; Class 3 = Mind and Body Crew; Class 4 = Dynamic Doers.*

Table S8: Item endorsement probabilities in each class (4-class model)

|  | Probability of High Endorsement of Category | | | |
| --- | --- | --- | --- | --- |
| PACE item | Dynamic Doers | Game and Gain Squad | Mind and Body Crew | Activity Free Adolescents |
| Cinema | 0.645 | **0.261** | **0.158** | **0.129** |
| Wating Sport | 0.544 | 0.41 | **0.085** | **0.053** |
| Music (e.g., orchestra) | **0.291** | **0.033** | **0.107** | **0.031** |
| Reading for Fun | **0.807** | **0.274** | 0.672 | **0.211** |
| Youth Clubs | 0.653 | 0.338 | **0.298** | **0.072** |
| Cultural activities (e.g., museums) | 0.553 | **0.039** | **0.093** | **0.024** |
| Religious activities | 0.511 | **0.186** | **0.280** | **0.155** |
| Arts and Crafts | **0.801** | **0.162** | **0.816** | **0.225** |
| Video games | **0.895** | **0.924** | **0.815** | 0.611 |
| Playing sports/exercise | **0.968** | **0.997** | **0.869** | 0.348 |
| Other creative hobbies | **0.923** | 0.559 | **0.915** | **0.234** |

*Item probabilities >0.7 or <0.3 are bolded to indicate a high degree of class homogeneity.*
